# Chemistry of the Nudibranch *Aldisa andersoni*: Structure and Biological Activity of Phorbazole Metabolites

**DOI:** 10.3390/md10081799

**Published:** 2012-08-20

**Authors:** Genoveffa Nuzzo, Maria Letizia Ciavatta, Robert Kiss, Véronique Mathieu, Helene Leclercqz, Emiliano Manzo, Guido Villani, Ernesto Mollo, Florence Lefranc, Lisette D’Souza, Margherita Gavagnin, Guido Cimino

**Affiliations:** 1 CNR, Instituto di Chimica Biomolecolare, Via Campi Flegrei 34, I-80078 Pozzuoli, Naples, Italy; Email: gnuzzo@icb.cnr.it (G.N.); emanzo@icb.cnr.it (E.M.); gvillani@icb.cnr.it (G.V.); emollo@icb.cnr.it (E.M.); mgavagnin@icb.cnr.it (M.G.); gcimino@icb.cnr.it (G.C.); 2 Laboratoire de Toxicologie, Faculté de Pharmacie, Université Libre de Bruxelles (ULB), Campus de la Plaine, Boulevard du Triomphe, 1050, Brussels, Belgium; Email: rkiss@ulb.ac.be (R.K.); vemathie@ulb.ac.be (V.M.); Helene.Leclercqz@ulb.ac.be (H.L.); 3 Service de Neurochirurgie, Hôpital Erasme, ULB, Route de Lennik, 1070 Brussels, Belgium; Email: fllefran@ulb.ac.be; 4 CSIR—National Institute of Oceanography, 403 004 Dona Paula, Goa, India; Email: lisette@nio.org

**Keywords:** *Aldisa andersoni*, nudibranch mollusc, structural elucidation, feeding-deterrence, growth inhibitory assays

## Abstract

The first chemical study of the Indo-Pacific dorid nudibranch *Aldisa andersoni* resulted in the isolation of five chlorinated phenyl-pyrrolyloxazoles belonging to the phorbazole series. Two new molecules, 9-chloro-phorbazole D and *N*1-methyl-phorbazole A, co-occurring with known phorbazoles A, B and D, have been characterized. Phorbazoles were found to be present mainly in the external part of the mollusc. The structures of the new compounds were determined by interpretation of spectroscopic data, mainly NMR and mass spectrometry and by comparison with the literature data. Evaluation of feeding-deterrence activity as well as *in vitro* growth inhibitory properties in human cancer cells was also carried out.

## 1. Introduction

Opisthobranchs are marine molluscs in which the shell is reduced or completely absent. They move slowly and their soft-bodies often present bright, attractive coloration, with multihued geometric patterns [1]. However, despite slow movements, the absence of physical attributes and gaudy livery, opisthobranchs have few documented predators. Field observations and proper ecological assays have demonstrated that they protect themselves by employing a series of defensive strategies including chemicals [2]. These compounds that play a fundamental role for their survival are usually small molecules derived from the diet, which can be accumulated in specialized anatomical parts of the animal, resulting in their unpleasant taste. In some species the presence of the *de novo* bio-synthesized defensive molecules has been clearly demonstrated [3,4]. The ability of opisthobranchs to select bioactive molecules from nature has resulted in an extraordinary library of compounds with intriguing framework, which are not found in their terrestrial counterparts and possess the potential as new pharmaceutical entities [5].

In our ongoing studies on the chemical ecology of marine opisthobranchs, we have examined the chemical content of six specimens of *Aldisa andersoni* collected off Muttom coast, India (Indian Ocean). *A. andersoni* is a dorid nudibranch (order Nudibranchia, infraorder Anthobranchia, superfamily Doridoidea) only recorded from Sri Lanka coast (not far from the place of our collection). The animal presents an oval body, 20–30 mm in length and a series of rows of low tubercles on the blue dorsum. The tubercles are separated by black areas of pigment, the upper part of the skin is crossed by a bright yellow saddle behind the rhinophores and other yellow marks on the dorsum [6]. These features appear to be indicative of aposematism (warning coloration), closely resembling the shape and the color pattern of unpalatable nudibranch species belonging to the genus *Phyllidia*, which are known to contain toxic isonitrile molecules [7]. Previous studies on different *Aldisa* species from Pacific and Mediterranean areas resulted in the finding of unusual steroids. In particular, two feeding-deterrent sterols featuring cholic acid side chain have been reported from *Aldisa cooperi * [8], whereas an unusual 24-norchol-4-ene-3,22-dione has been isolated from *Aldisa smaragdina* [9]. It seems that the source of steroids in *Aldisa cooperi* should be the sponge *Anthoarcuata graceae* but the animal is able to modify the inactive cholestenone into two active compounds. On the other hand, the absence of the steroid isolated from *A. smaragdina* in its prey, the sponge *Phorbas fictitius*, suggests the modification of a suitable precursor or a biosynthetic origin.

The chemistry of *A. andersoni* has not been studied previously. We found evidence of the presence of chlorinated phenyl pyrrolyloxazoles in the ether extract of the mollusc. These molecules were found to be mainly concentrated in the external part of the mollusc even though they were also detected in the internal glands. Two new molecules, 9-chloro-phorbazole D (**1**) and *N*1-methyl-phorbazole A (**2**), co-occurring with the known related phorbazoles A, B and D have been isolated and characterized from this mollusc. Phorbazoles have been reported to date only from the sponge *Phorbas* aff. *clathrata* [10,11,12]. The origin of these metabolites in *A. andersoni* could be ascribed to a diet based on *Phorbas* sponges although *de novo* biosynthesis could not be excluded.

## 2. Results and Discussion

Six specimens of *A. andersoni* were caught by scuba diving off Muttom coast (South India) during November 2009, were immediately frozen at −20 °C and transferred to the laboratory. Unfortunately no plausible prey was observed in the same area. Anatomical dissection of the body of nudibranchs was precluded due to the small size and the following procedure was used to obtain the distinct extracts of external and internal parts. First, the whole animals were immersed in acetone (20 mL) and submitted to ultrasound vibration for a few minutes. By this procedure only the metabolites present in the skin (external part) were extracted. The solvent was removed and the animals were crushed by a pestle and again treated with acetone (20 mL × 3), to extract the digestive gland contents (internal part). After evaporation of acetone, the aqueous residue of both the extracts was partitioned between Et_2_O (50 mL × 4) and H_2_O. The two ether extracts were analyzed by Thin Layer Chromatography (TLC) developed in the solvent system (CHCl_3_/CH_3_OH, 8:2). Both exhibited similar metabolite pattern with a series of UV-visible spots significantly more concentrated in the external extract. This extract was then directly submitted to RP-HPLC purification (MeOH/H_2_O gradient) which resulted in the novel compounds **1** and **2** along with known phorbazoles A, B and D, that were identified by comparison of their spectroscopic data with the literature values [10] (see Experimental Section for details).

Analysis of NMR spectra of compounds **1** and **2** revealed a close structural relationship with known phorbazoles however indicating differences in the substitution pattern.

**Figure 1:**
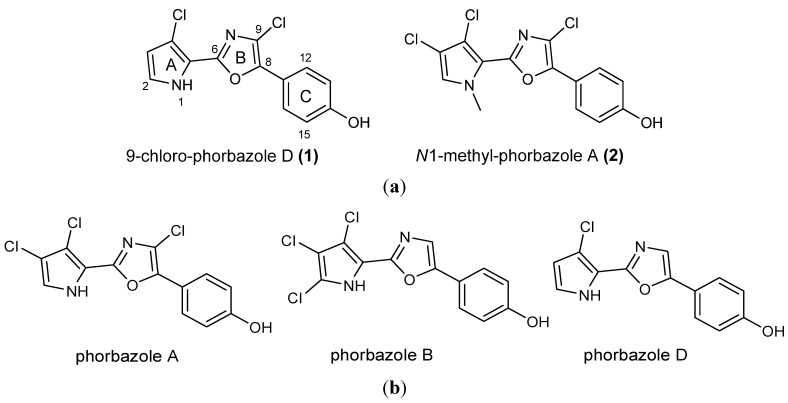
(**a**) Structures of novel phorbazoles isolated from the external part of *A. andersoni*; (**b**) Structures of known phorbazoles co-occurring in the same extract.

**Figure 2:**
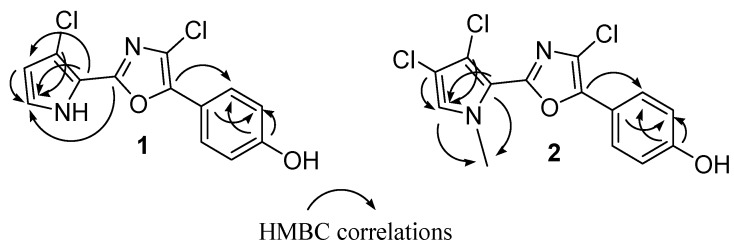
Key HMBC correlations for phorbazoles **1** and **2**.

Compound **1** had a molecular formula C_13_H_8_Cl_2_N_2_O_2_ deduced by LCMS (*m/z* 295 [M + H]^+^) and HRESIMS (*m/z* 316.9875 [M + Na]^+^), that showed a characteristic cluster for two chlorine atoms. The ^1^H NMR spectrum of **1 **exhibited only four sp^2^ signals at δ 6.27 (1 H, d, *J* = 2.8 Hz, H-3), 6.93 (2H, d, *J* = 8.7 Hz, H-13 and H-15), 6.97 (1H, d, *J =* 2.8 Hz, H-2) and 7.81 (2H, d, *J* = 8.7 Hz, H-12 and H-16), according to two separated spin systems in the disubstituted pyrrole unit (ring A) and 1,4 disubstituted benzene ring (ring C). This implied that the two chlorine atoms were located in both the pyrrole and oxazole rings. Consistent with this, the ^13^C NMR spectrum contained 11 sp^2^ carbon signals in the range of 111.8–159.6 ppm (Table 1). The 2D NMR experiments aided us to fully characterize the molecule. In particular, a series of HMBC experiments, recorded with *J* = 5 Hz and *J* = 10 Hz, were crucial to attribute the quaternary carbons and to assign the structure as depicted in Figure 1. In fact, diagnostic correlations were observed between C-8 (δ_C_ 144.2) and H-12/H-16, and between C-6 (δ_C_ 153.7) to H-2 indicating the connection of the oxazole ring to both 1,4-disubstituted benzene and pyrrole. Expected HMBC correlations were also observed between C-5, C-4 and C-3 to H-2 (Figure 2). Comparison of NMR data with those recorded in the same solvent for phorbazoles (see Experimental section) confirmed the suggested structure. Compound **1** possessed one more chlorine atom on the oxazole ring with respect to phorbazole D, thus it was named 9-chloro-phorbazole D.

The ESIMS analysis of compound **2** showed peaks at *m/z* 343 [M + H]^+^ with the typical cluster due to the presence of three chlorine atoms. The HRESIMS spectrum indicated the molecular formula C_14_H_9_Cl_3_N_2_O_2_ as deduced from the sodiated peak at *m/z* 364.9634 [M + Na]^+^. The NMR spectra of **2** (Table 1) showed proton and carbon resonances very similar to those of phorbazole A (see Experimental Section and [10]), indicating the same substitution pattern in the heterocyclic rings. The only difference was in the presence of a 3H signal resonating at δ_H_ 4.00 (δ_C_ 38.4), which was attributed to a methyl linked at the pyrrole nitrogen. Thus compound **2** was suggested to be *N*1-methyl-phorbazole A. Significant HMBC correlations were observed between the *N*1-Me and both C-2 (δ_C_ 125.2) and C-5 (δ_C_ 115.3), in agreement with the depicted structure (Figure 2). Treatment of phorbazole A with MeI (see Experimental Section) gave a compound identical in all respects with compound **2**, definitively confirming the structure.

**Table 1:** ^1^H (400/600 MHz) and ^13^C (75/150 MHz) NMR data ^a^ of compounds **1** and **2 **in CD_3_OD.

Position	1		2		
	δ_C_ mult.	δ_H_ ( *J* in Hz)		δ_C_ mult.	δ_H_ ( *J* in Hz)
2	122.8, CH	6.97, d (2.8)		125.2, CH	7.10, s
3	111.8, CH	6.27, d (2.8)		114.7, C	
4	111.8, C			112.4, C	
5	115.8, C			115.3, C	
6	153.7, C			152.3, C	
8	144.2, C			144.2, C	
9	116.6, C			117.9, C	
11	119.4, C			119.1, C	
12,16	127.7, CH	7.81, d (8.7)		127.8, CH	7.81, d (8.7)
13,15	116.5, CH	6.93, d (8.7)		116.9, CH	6.93, d (8.7)
14	159.6, C			159.8, C	
*N*1-Me	122.8, CH			38.4, CH_3_	4.00, s

^a^ assignment made by ^1^H-^1^H COSY, HSQC, and HMBC (*J* = 10 and 5 Hz) experiments.

**Figure 3:**
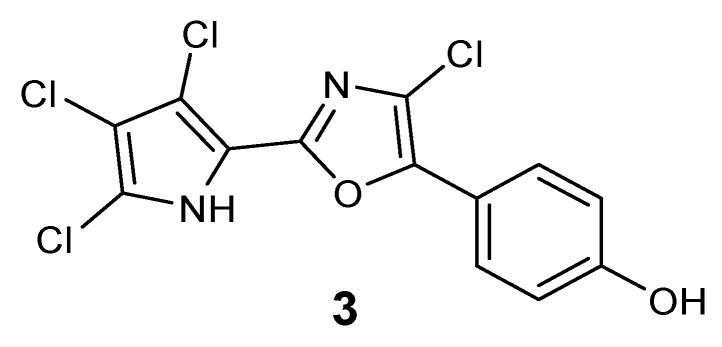
Plausible structure of 2-chloro-phorbazole A (**3**).

The presence of a very minor phorbazole compound was detected in the analyzed ethereal extract. This metabolite was isolated in very small amount which was insufficient for a detailed spectroscopic characterization. However, the ^1^H NMR and ESIMS spectra were recorded and indicated the presence of a structure in which all positions in both the oxazole and pyrrole rings were substituted with chlorine atoms. In fact, a protonated molecular peak at *m/z* 363 [M + H]^+^ with a cluster consistent with the presence of four chlorine atoms was observed in the ESIMS spectrum whereas the ^1^H NMR spectrum exhibited only two doublet signals due to the 4-hydroxyphenyl moiety (see Experimental Section). The compound could be tentatively identified as 2-chloro-phorbazole A, as depicted in Figure 3. 

**Figure 4:**
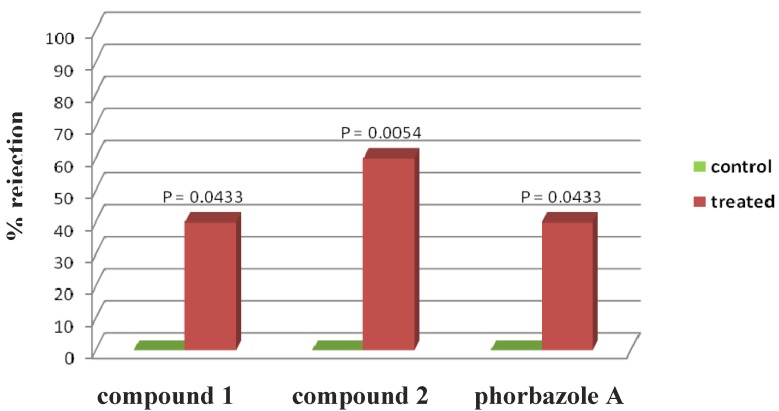
*Palaemon elegans* alimentary response. The zero concentration was defined as control, and significant differences in the consumption of treated *vs*. control pellets have been evaluated by one-tailed Fisher’s exact test (α = 0.05, *n* = 10 for each bar).

Phorbazole compounds were particularly concentrated in the most exposed external part of the nudibranch, suggesting their involvement in chemical defense. Selected phorbazoles were thus tested in the feeding deterrence assay (see Experimental Section) against the trophic generalist shrimp *Palaemon elegans* [13]. Even though paucity of material prevented us from obtaining dose response curves for each compound, compound **1**, compound **2** and phorbazole A resulted in significant feeding deterrence at a concentration of 1.0 mg/mL (Figure 4). This finding supports that *A. andersoni*, together with similarly colored phyllidiid nudibranchs, are members of a Müllerian mimetic circle in which different unpalatable species had an advantage from becoming similar to others [14], sharing conspicuous visual signals that predators rapidly learn to avoid.

Preceding studies have indicated that marine invertebrates produce bioactive natural products that may be useful for developing new drugs. [15] By exploring untapped geographical sources and/or novel groups of organisms one can maximize the search for new marine drugs to treat human diseases. Although immuno-modulatory activity on previously reported phorbazoles has been claimed by the authors [10], no manuscript has appeared on the subject. In this regard, the *in vitro* growth inhibitory activity of phorbazoles has been investigated on a panel of five human cancer cell lines, which included two cell lines displaying actual sensitivity to pro-apoptotic stimuli, *i.e.*, the MCF-7 mammary adenocarcinoma [16,17] and the Hs683 oligodendroglioma [17,18] and three cell lines displaying various levels of resistance to pro-apoptotic stimuli, *i.e.*, the A549 non-small-cell lung cancer [19] (NSCLC), the SKMEL-28 melanoma [20] and the U373 glioblastoma [17,18]. The MTT colorimetric assay [16,17,20] was employed to measure cell growth. The data obtained is illustrated in Table 2.

**Table 2:** Determination of the IC_50_ (µM) *in vitro* growth inhibitory concentration by means of the MTT colorimetric assay in five human cancer cell lines.

Compounds *versus* Cancer Cell Lines (IC_50_; µM)	1	2
	(9-chloro-phorbazole D)	(*N*1-methyl-phorbazole A)
A549 (NSCLC)	29	34
MCF-7 (breast cancer)	18	25
SKMEL-28 (melanoma)	22	29
Hs683 (oligodendroglioma)	25	25
U373 (glioblastoma)	19	19
Mean ± SEM	22 ± 2	26 ± 2

The human cancer cell lines include the A549 NSCLC (DSMZ code ACC107), the MCF-7 breast adenocarcinoma (Deutsche Sammlung von Mikroorganismen und Zellkulturen (DSMZ) code ACC115, the SKMEL-28 melanoma (ATCC code HTB-72), the Hs683 oligodendroglioma (American Type Culture Collection (ATCC) code HTB-138), the U373 glioblastoma (European Collection of Cell Culture (ECACC) code 89081403) cell lines.

The data shows that compounds **1** and **2** display similar *in vitro* growth inhibitory activity in the five human cancer cell lines under study and that the growth inhibitory activity of these two compounds is not modified as whether the cancer cells display actual sensitivity (MCF-7; Hs683) as opposed to various levels of resistance (A549; SKMEL-28; U373) to pro-apoptotic stimuli. The *in vitro* growth inhibitory activity displayed by **1** and **2** are of the same range, or even higher, than those displayed by carboplatin [21] and temozolomide [22], which are the two compounds largely used to treat patients with aggressive cancers [23,24].

**Figure 5:**
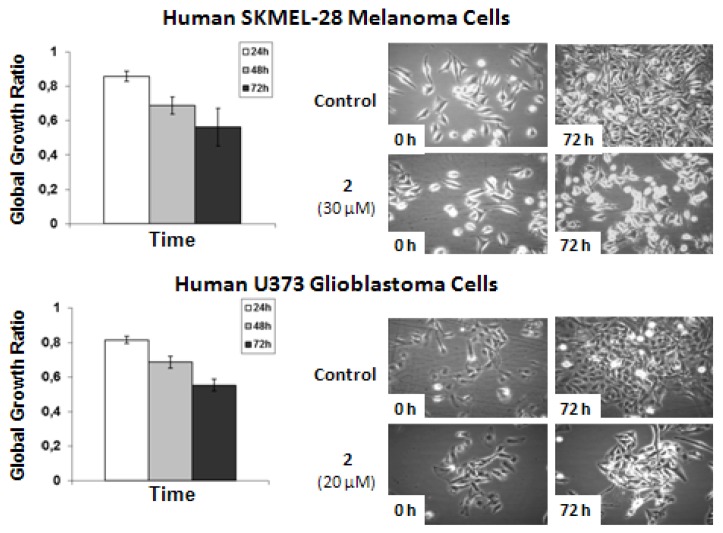
Quantitative videomicroscopy analyses of **2**-induced effects on SKMEL-28 melanoma and U373 glioblastoma cells.

Computer-assisted phase contrast microscopy [25,26] (quantitative videomicroscopy) was then used to determine as to whether the *in vitro* growth inhibitory activity observed for **2** (for which sufficient amounts were available) was related or not to the cytostatic *versus* cytotoxic effects. The quantitative videomicroscopy analyses confirmed the *in vitro* growth inhibitory activity of *N*1-methyl-phorbazole A (**2**) as initially revealed by the MTT colorimetric assay (Table 2) and as illustrated below in Figure 5. 

The data shows that **2**-related *in vitro* anticancer activity mainly relates to cytostatic effects in both human SKMEL-28 melanoma and U373 glioblastoma cells. The concentrations used to analyze the effects of **2** by means of quantitative videomicroscopy were those obtained by means of the MTT colorimetric assay, *i.e.*, 30 μM for SKMEL-28 melanoma cells and 20 μM for U373 glioblastoma cells (Table 2). These MTT test-related IC_50_ concentrations (Table 2) also turned out to decrease by ~50% the global growth activity in U373 glioblastoma and SKMEL-28 melanoma cells under quantitative videomicroscopy monitoring (Figure 5).

## 3. Experimental Section

### 3.1. General Experimental Procedures

Optical rotations were measured using a Jasco DIP 370 digital spectropolarimeter. IR spectra were measured on a Biorad FTS 155 FTIR spectrophotometer. 1D- and 2D-NMR spectra were recorded on Bruker Avance-400 (400.13 MHz) and Bruker DRX-600 equipped with TXI CryoProbeTM in CD_3_OD and DMSO-*d*_6_ (shifts are referenced to the solvent signal at δ 3.34 and 2.50, respectively) and ^13^C NMR spectra were recorded on Bruker DPX-300 (75 MHz) and Bruker DRX-600 (150 MHz) spectrometers (δ values are reported to CD_3_OD, 49.0 ppm and to DMSO-*d*_6_ at 39.5 ppm). HRESIMS measurements were carried out on Micromass Q-TOF micro; HPLC Shimadzu liquid chromatograph LC-10AD was equipped with an UV SPD-10A wavelength detector and performed with a semipreparative column RP-18 (Supelco, 250 mm × 46 mm, 5 μm); TLC plates (KieselGel 60 F254) were from Merck. Solvents for chromatography were HPLC grade hence were used without further purification.

### 3.2. Sample Collection

Six specimens of *Aldisa andersoni* were collected in November 2009 from the Indian Ocean by G.V. and transferred to the laboratory. The nudibranchs (average size 25 mm) were identified by Dr. Juan Lucas Cervera (University of Cadiz). 

### 3.3. Extraction and Purification

The frozen molluscs *A. andersoni* were treated, in order to obtain extracts of the mantle and the internal glands, separately. The external extract was obtained by simply soaking the whole animals in acetone bath (20 mL) for few minutes, whereas the internal extract was obtained by grinding the animal after sonication. The extracts were concentrated *in vacuo* and the aqueous residues were then fractionated between H_2_O and Et_2_O (4 × 50 mL). The Et_2_O portions were evaporated under reduced pressure to give 15 mg from the external and 62 mg from the internal parts of the nudibranchs. The external part extract was directly purified by C18 reversed-phase HPLC using a Supelco RP-18 column (250 × 4.60 mm) with a linear gradient from MeOH/H_2_O 70:30 to MeOH 100% in 40 min (flow 1 mL/min) to afford in order of decreasing polarity phorbazole D (*t*_R_ 7.4 min, 0.7 mg), 9-chloro-phorbazole D (**1**) (*t*_R_ 11.8 min, 4.9 mg), phorbazole A (*t*_R_ 19.2 min, 4.0 mg), phorbazole B (*t*_R_ 21.0 min, 2.5 mg), *N*1-methyl-phorbazole A (**2**) (*t*_R_ 28.9 min, 1.1 mg), and 2-chloro-phorbazole A (**3**) (*t*_R_ 31.2 min, <0.5 mg). 

### 3.4. 9-Chloro-phorbazole D *(**1**)*

Yellow powder (32.7%); UV (MeOH) λ_max_ (log ε) 208 (6.90), 256 (6.80), 334 (7.01); IR (liquid film) ν_max_ 1614, 1506, 1449, 1406, 1278, 1176, 837 cm^−1^; ^1^H NMR and ^13^C NMR CD_3_OD, see Table 1, ^1^H NMR in DMSO-*d*_6_: δ_H_ 7.73 (d, ^3^*J*_H,H_ = 8.4 Hz, 2H, H-12 and H-16), δ_H_ 7.07 (d, ^3^*J*_H,H_ = 2.9 Hz, 1H, H-2), δ_H_ 6.94 (d, ^3^*J*_H,H_ = 8.4 Hz, 2H, H-13 and H-15), δ_H_ 6.31 (d, ^3^*J*_H,H_ = 2.9 Hz, 1H, H-3); ^13^C NMR in DMSO-*d*_6_: δ_C_ 158.3 (C-14), δ_C_ 151.7 (C-6), δ_C_ 142.1 (C-8), δ_C_ 126.2 (C-12 and C-16), δ_C_ 122.5 (C-2), δ_C_ 122.3 (C-9), δ_C_ 116.9 (C-11), δ_C_ 116.0 (C-13 and C-15), δ_C_ 114.7 (C-5), δ_C_ 113.2 (C-4), δ_C_ 110.6 (C-3). HRESIMS [M + Na]^+^ *m/z* 316.9875 (calcd for C_13_H_8_Cl_2_N_2_O_2_, 316.9861). 

### 3.5. *N*1-Methyl-phorbazole A *(**2**)*

Yellow powder (7.3%); ^1^H NMR and ^13^C NMR in CD_3_OD, see Table 1, ^1^H NMR in DMSO-*d_6_*: δ_H_ 7.71 (d, ^3^*J*_H,H_ = 8.8 Hz, 2H, H-12 and H-16), δ_H_ 7.42 (s, H-2), δ_H_ 6.94 (d, ^3^*J*_H,H_ = 8.8 Hz, 2H, H-13 and H-15), δ_H_ 3.92 (s, 1-*N*-Me); ^13^C NMR in DMSO-*d*_6_: δ_C_ 157.6 (C-14), δ_C_ 151.3 (C-6), δ_C_ 142.3 (C-8), δ_C_ 126.1 (C-12 and C-16), δ_C_ 124.2 (C-2), δ_C_ 122.4 (C-9), δ_C_ 116.1 (C-11), δ_C_ 115.7 (C-13 and C-15), δ_C_ 115.3 (C-5), δ_C_ 111.2 (C-4), δ_C_ 109.0 (C-3), δ_C_ 37.5 (*N*1-Me). HRESIMS [M + Na]^+^ *m/z* 364.9634 (calcd for C_14_H_9_Cl_3_N_2_O_2_, 364.9627). 

### 3.6. Methylation of Phorbazole A

An aliquot of phorbazole A (1 mg) was treated with 1 mL of MeI and Na_2_CO_3_ (12 mg) in 500 μL of anhydrous acetone stirring at r.t. for 3 h. As reaction reached the completion, the acetone was removed under reduced pressure, and the reaction mixture was loaded into a SiO_2_ column eluting with CHCl_3_/MeOH 8:2, to obtain 0.8 mg of a compound identical with the natural compound **2 **by NMR and MS.

### 3.7. 2-Chloro-phorbazole A *(**3**)*

LCMS (*m/z* 363 [M + H]^+^); ^1^H NMR in CD_3_OD: δ_H_ 7.82 (d, ^3^*J*_H,H_ = 8.7 Hz, 2H, H-12 and H-16), δ_H_ 6.94 (d, ^3^*J*_H,H_ = 8.7 Hz, 2H, H-13 and H-15).

### 3.8. NMR Data of Phorbazole A in CD_3_OD

^1^H NMR (recorded at 600 MHz) δ_H_ 7.80 (d, ^3^*J*_H,H_ = 8.7, 2H, H-12 and H-16), δ_H_ 7.08 (s, 1H, H-2), δ_H_ 6.92 (d, ^3^*J*_H,H_ = 8.7, 2H, H-13 and H-15); ^13^C NMR δ_C_ 159.7 (C-14, C), δ_C_ 152.8 (C-6, C), δ_C_ 144.6 (C-8, C), δ_C_ 127.8 (C-12 and C-16, CH), δ_C_ 124.4 (C-9, C), δ_C_ 120.2 (C-2, CH), δ_C_ 119.1 (C-5 and C-11, C), δ_C_ 116.8 (C-13 and C-15, CH), δ_C_ 113.9 (C-3 or C-4, C), δ_C_ 113.6 (C-4 or C-3, C).

### 3.9. NMR Data of Phorbazole B in CD_3_OD

^1^H NMR (recorded at 600 MHz) δ_H_ 7.51 (d, ^3^*J*_H,H_ = 8.0, 2H, H-12 and H-16), δ_H_ 7.29 (s, 1H, H-9), δ_H_ 6.82 (d, ^3^*J*_H,H_ = 8.0, 2H, H-13 and H-15); ^13^C NMR δ_C_ 159.3 (C-14, C), δ_C_ 152.1 (C-8, C), δ_C_ 155.5 (C-6, C), δ_C_ 126.8 (C-12 and C-16, CH), δ_C_ 121.1 (C-9, C), δ_C_ 120.6 (C-11, C), δ_C_ 117.8 (C-5 and C-2, C), δ_C_ 116.8 (C-13 and C-15, CH), δ_C_ 111.9 (C-3 and C-4, C).

### 3.10. NMR Data of Phorbazole D in CD_3_OD

^1^H NMR (recorded at 600 MHz) δ_H_ 7.64 (d, ^3^*J*_H,H_ = 8.0, 2H, H-12 and H-16), δ_H_ 7.36 (s, 1H, H-9), δ_H_ 6.95 (d, *^3^J*_H*,* H_ = 2.0 Hz, 1H, H-2), δ_H_ 6.90 (d, ^3^*J* = 8.0, 2H, H-13 and H-15), δ_H_ 6.26 (d, ^3^*J*_H,H_ = 2.0 Hz, 1H, H-3); ^13^C NMR δ_C_ 159.5 (C-14, C), δ_C_ 155.2 (C-6, C), δ_C_ 152.2 (C-8, C), δ_C_ 126.2 (C-12 and C-16, CH), δ_C_ 121.8 (C-2, CH), δ_C_ 120.5 (C-9, CH), δ_C_ 119.8 (C-11, C), δ_C_ 116.3 (C-13 and C-15, CH), δ_C_ 111.1 (C-3, CH), C-4 and C-5 n.d.

### 3.11. Feeding-Deterrence Assay

The compounds were tested for their feeding deterrence activity against the generalist shrimp *Palaemon elegans* (Rathke, 1837). Assays were performed as described in Mollo [13], by using food pellets treated with compounds **1** and **2**, and phorbazole A, at concentrations of 1.0 mg/mL. Each pure compound, dissolved with 0.5 mL of acetone to give the desired final concentration, was added to a mixture of lyophilized squid mantle, alginate, purified sea sand and mixed. After evaporation of the solvent, distilled water and a drop of red food color were added to make a final volume of 1.0 mL. The paste was hardened into a 0.25 M CaCl_2_ solution (two minutes) and cut into 10 mm long strips. Accordingly, controls were prepared with acetone only (zero concentration). Shrimps were collected along the coast of Pozzuoli, Italy, and habituated to the control food for a week before experiments. After three days of total fasting, ten randomly picked shrimps were assayed as a series of individual replicates for each concentration and the control (*n* = 10 for each series). Shrimps were placed individually into plastic beakers filled with seawater. A colored food strip was given to each shrimp, and shrimps were not re-used. Control and treatments were carried out in parallel. The presence of a red spot visible by transparency in the gastric mill and the stomach of the shrimps after 30 min was considered as an acceptance of the food, while the absence of the spot gave a rejection response. Statistical analysis between treatments and controls was performed using the one-tailed Fisher-Exact test, which is traditionally used with relatively small samples, with α = 0.05 as significant level. After the experiments, the shrimps were returned in field to the same location they were collected.

### 3.12. Determination of *in Vitro* Anticancer Activity

The histological types and origins of the five cancer cell lines that were used for the MTTcolorimetric assay are detailed in Table 2. The cells were cultured in RPMI (Lonza, Verviers, Belgium) medium supplemented with 10% heat inactivated foetal calf serum (Lonza). All culture media were supplemented with 4 mM glutamine, 100 µg/mL gentamicin, and 200 U/mL penicillin and 200 µg/mL streptomycin (Lonza). The overall growth level of the human cancer cell lines was determined using a colorimetric MTT (3-[4,5-dimethylthiazol-2yl]-diphenyl tetrazolium bromide, Sigma, Belgium) assay as detailed previously [16,17,20,21]. Six replicates of each experimental condition were performed.. Thus, this procedure enables the concentration of **1** and **2** that decreased by 50% the growth of each cell line after having cultured it with the compound of interest for 72 h (the IC_50_ index in μM) to be determined.

### 3.13. Computer-Assisted Phase Contrast Microscopy (Quantitative Videomicroscopy)

The direct visualization of compound-induced effects in terms of cytotoxicity *versus* cytostaticity in human SKMEL-28 melanoma and U373 glioblastoma cells was achieved as detailed in literature [25,26]. Briefly, the quantitative videomicroscopy approach enables an image of the bottom of the seeded flask to be taken every four minutes over the course of a 72 h-observation period. Thus, 1080 digitized images are available for each experimental condition, which were run in triplicates. A global growth ratio (the GGR index) was calculated for **2**, resulting in a value that can be directly compared to the MTT assay-determined IC_50_ value (Table 2). First, the global growth (GG) is calculated in each control and in each treated condition by dividing the number of cells on the last image by the number of cells on the first image. The GGR index was thus obtained for **2** by dividing the GG values calculated for treated SKMEL-28 or U373 cells by the GG values calculated for the control (the data are presented as mean ± SEM values). For example, a GGR value of 0.5 for **2** at a given experimental time means that this compound decreased by 50% (1.0 − 0.5 = 0.5) the global growth of the considered cancer cell population as compared to control. 

## 4. Conclusions

Phorbazoles belong to a family of compounds first isolated by Kashman *et al.* from the sponge *Phorbas* aff. *clathrata* [10]. These metabolites have a unique structure in common consisting of a pyrrole, an oxazole and a phenol ring differing in the number and position of chlorine atoms. The dietary origin, most likely from a *Phorbas* sponge, of phorbazoles in *A. andersoni*, could be assumed on the basis of their presence in the digestive organs. However, no sponges of this genus were observed in the area of collection of the nudibranch population. Moreover, the presence of such chemicals particularly concentrated in the external part of the nudibranch suggested their involvement in chemical defense against potential predators, which has been confirmed by feeding deterrence assays. Finally, the *in vitro* cytostatic effect of selected phorbazoles in a small panel of tumor cells has been measured for the first time.

## Declare

In memory of Professor Ernesto Fattorusso.
